# Identification of a Major QTL That Alters Flowering Time at Elevated [CO_2_] in *Arabidopsis thaliana*

**DOI:** 10.1371/journal.pone.0049028

**Published:** 2012-11-21

**Authors:** Joy K. Ward, Debosree Samanta Roy, Iera Chatterjee, Courtney R. Bone, Clint J. Springer, John K. Kelly

**Affiliations:** Department of Ecology and Evolutionary Biology, University of Kansas, Lawrence, Kansas, United States of America; The Australian National University, Australia

## Abstract

**Background:**

The transition from vegetative to reproductive stages marks a major milestone in plant development. It is clear that global change factors (e.g., increasing [CO_2_] and temperature) have already had and will continue to have a large impact on plant flowering times in the future. Increasing atmospheric [CO_2_] has recently been shown to affect flowering time, and may produce even greater responses than increasing temperature. Much is known about the genes influencing flowering time, although their relevance to changing [CO_2_] is not well understood. Thus, we present the first study to identify QTL (Quantitative Trait Loci) that affect flowering time at elevated [CO_2_] in *Arabidopsis thaliana*.

**Methodology/Principal Findings:**

We developed our mapping population by crossing a genotype previously selected for high fitness at elevated [CO_2_] (SG, Selection Genotype) to a Cape Verde genotype (Cvi-0). SG exhibits delayed flowering at elevated [CO_2_], whereas Cvi-0 is non-responsive to elevated [CO_2_] for flowering time. We mapped one major QTL to the upper portion of chromosome 1 that explains 1/3 of the difference in flowering time between current and elevated [CO_2_] between the SG and Cvi-0 parents. This QTL also alters the stage at which flowering occurs, as determined from higher rosette leaf number at flowering in RILs (Recombinant Inbred Lines) harboring the SG allele. A follow-up study using *Arabidopsis* mutants for flowering time genes within the significant QTL suggests MOTHER OF FT AND TFL1 (MFT) as a potential candidate gene for altered flowering time at elevated [CO_2_].

**Conclusion/Significance:**

This work sheds light on the underlying genetic architecture that controls flowering time at elevated [CO_2_]. Prior to this work, very little to nothing was known about these mechanisms at the genomic level. Such a broader understanding will be key for better predicting shifts in plant phenology and for developing successful crops for future environments.

## Introduction

The transition within plants from vegetative to reproductive stages can have major implications for fitness, evolutionary processes, and species interactions [1]. For short-lived annual species, the onset of reproduction is particularly critical, since it also marks the early stages of senescence [2]. The implications of flowering time are also context-dependent, and selective pressures on this trait can vary depending on local conditions, and these responses can be further modified through global change drivers (e.g., increasing [CO_2_] and temperature). For example, if flowering time is overly delayed, reproduction may be incomplete or may fail all together, particularly in regions where cold temperatures or drought punctuate the end of the growing season. On the other hand, if the transition to flowering is too rapid, the full length of the growing season may not be utilized for maximal gain of carbon resources that are essential to maximize reproduction [1], [3], [4]. Under either scenario, pollinator services may become decoupled from flowering, producing negative effects at higher trophic levels [5] (but also see [6], [7]).

Global change factors are known to have already influenced flowering time in a number of species, and changes in these factors are expected to have an even greater impact in the future [8]. Temperature effects have garnered the most attention, whereby increasing temperatures are generally found to accelerate flowering times in some experimental studies that manipulate temperature (but see [9]), with even greater responses in field studies documenting changes in flowering times over the past several decades to a millennium [10]. The direct effects of rising atmospheric [CO_2_] on flowering time have received much less attention, and are not as well understood at the mechanistic level, but can be as large or larger in magnitude as temperature effects [11]. In addition, rising [CO_2_] occurs on a global scale, whereas temperature change varies at the regional level. In a literature survey [11], we found that 57% of tested wild species and 62% of crop species (mostly annuals) exhibited altered flowering times when grown at ≈350 versus ≈700 ppm CO_2_ (with temperature being constant). The extreme responses at elevated [CO_2_] ranged from accelerations of 60 days to delays of 16 days, depending on the species. Furthermore, the effects of elevated [CO_2_] on flowering time have been shown to vary *within* species as well. For example, *Arabidopsis thaliana* (hereafter *Arabidopsis*) exhibits delayed, accelerated, and neutral responses to elevated [CO_2_], depending on the genotype [11]. Pronounced inter- and intra-specific effects of CO_2_ on flowering times indicate that there is strong potential for community and genetic shifts in response to rising [CO_2_], and illustrate that the influence of CO_2_ on flowering time can no longer be ignored in the realm of global change/phenology studies [12].

Understanding the genetic basis for CO_2_ effects on flowering time is critical for predicting plant developmental patterns of the future. Unfortunately, little is known about the underlying mechanisms controlling this process. Early work suggested that photoperiod requirements play a role in modulating the effects of elevated [CO_2_] on flowering time. More specifically, Reekie and colleagues (1994) [13] found that four short-day annual species delayed flowering between 350 and 1000 ppm CO_2_, whereas four long-day annual species accelerated or exhibited no change in flowering between those treatments. Later work with mutants of *Arabidopsis* for genes in the photoperiod pathway suggested that CO_2_ levels interact with genes that sense and transduce light signals [14]. These responses suggest “cross-talk” between the photoperiod pathway and other pathways responding to elevated [CO_2_], possibly through sugar signaling ([11], [14]. In other studies, accelerated flowering at elevated [CO_2_] was determined to be a result of faster growth rates that allowed plants to reach the minimum size for reproduction faster, whereas delayed flowering was much more difficult to explain [15]. Along this line, soybean grown under FACE (Free-Air CO_2_ Enrichment) conditions exhibited delayed reproduction, even though elevated [CO_2_] warmed canopy temperatures as a result of reduced stomatal conductance; this is an effect that would have been expected to accelerate developmental timing rather than delay it [16], indicating that there are unknown mechanisms at play here.

In past work, we investigated delayed flowering at elevated [CO_2_] using a genotype of *Arabidopsis thaliana* that was selected for high fitness at elevated [CO_2_] over five generations (named SG for “Selection Genotype”; [17], [18]). SG exhibited the most pronounced response to selection among many selected plants, and is adapted to future high [CO_2_] conditions. SG exhibits altered expression of floral-initiation genes at elevated [CO_2_], whereby *FLC (FLOWERING LOCUS C)*, a strong repressor of flowering [19], remains highly expressed in SG grown at elevated [CO_2_] (700 ppm), producing major delays in flowering time and with flowering occurring at a much larger plant size. In contrast, at current [CO_2_] this gene exhibits the normal decreasing expression pattern through time, allowing flowering to occur in a timely manner and at a typical plant size [18]. This was the first demonstration of a CO_2_ influence on the expression of floral initiation genes, and this work targeted CO_2_ effects on flowering time to factors associated with the autonomous pathway. Moreover, this work showed that in addition to growth temperature, day length, and cold requirements, CO_2_ also influences the expression of floral-initiation genes and ultimately flowering time.

In order to better predict the effects of rising [CO_2_] on flowering time, a broader genomic understanding is needed. To our knowledge, we present the first QTL analysis of flowering time responses to elevated [CO_2_]. This work involved a parental cross between the SG genotype that delays flowering at elevated [CO_2_] [17], [18], and genotype Cvi-0 from Cape Verde that shows neutral responses to elevated [CO_2_] for flowering time. In this study, we worked to identify QTL that influence flowering time at elevated [CO_2_] in order to understand the underlying genetic architecture controlling this process and to improve our knowledge of QTL that are most relevant to future global change.

## Methods

### Development of Recombinant Inbred Lines (RILs)

We initially conducted a parental cross between the SG genotype, which had been previously selected for high fitness at elevated [CO_2_] [17], [18], and genotype Cvi-0 (CS902) that originated from Cape Verde (maintained at the *Arabidopsis* Biological Resource Center, The Ohio State University). These two parents exhibit differences in flowering time in response to CO_2_, with SG showing delayed flowering at elevated [CO_2_] and Cvi-0 showing neutral responses [4], [20]. The parental cross with Cvi-0 as father and SG as mother produced a single F_1_. We self-fertilized this F_1_ to produce a large progeny population with each F_2_ founding a distinct lineage. Through 5 more successive generations, we produced 189 F_7_ RIL lines by selfing and single seed descent (predicted homozygosity is 98.4%). These F_7_ RIL lines were then used for the phenotypic analysis of this QTL study.

### Identification of SNPs and Genotyping of RILs

**Figure 1:**
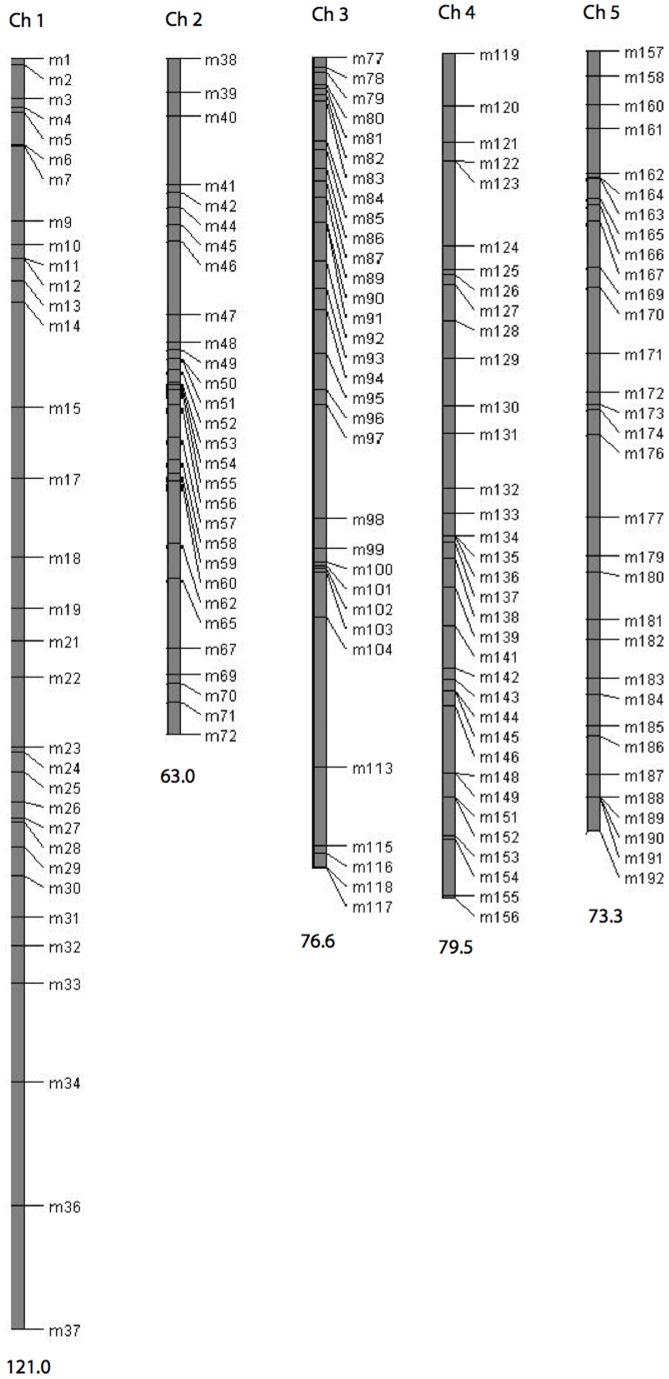
Linkage map showing the distribution of SNP markers across the five chromosomes used in our QTL study. The markers associated with particular flowering time genes are identified in Table S1.

Our overall strategy was to first identify a set of SNPs (single nucleotide polymorphisms) within the mapping population and then to genotype each RIL at these markers. To identify SNPs, full genome sequences from both parents were compared. The Cvi-0 genome had been previously sequenced by [21]. We used the Illumina Genome Analyzer II to sequence the SG genome to approximately 8X coverage (single end 36 bp reads; the reactions were conducted by Illumina, Inc., San Diego, CA). For sequencing purposes, high quality DNA was first extracted from SG using standard phenol-chloroform extraction with subsequent precipitation with isopropanol and storage in TE buffer (10 mM Tris, pH 7.5, 1 mM EDTA). We aligned reads to both the Columbia reference genome sequence (available on the *Arabidopsis* TAIR website: www.arabidopsis.org/) and the Cvi-0 genome using the MAQ alignment program (now replaced by BWA, [22]). Following alignment, we identified sites that were polymorphic in our RIL set from the SG versus Cvi-0 comparison. SNP calling was initially done using SAMTOOLs [23]. We then directly inspected the aligned reads for SNPs in selected regions to confirm high coverage and base call confidence. Locations of 192 genotyped SNPs were selected to span all five chromosomes of *Arabidopsis*. Of these, 47 markers were identified in the genomic vicinities (within 10 kb) of flowering time genes ([24]; an updated list was provided by Purugganan, M., *per comm.*). The remaining loci (145 in total) were chosen to provide approximately even coverage across the chromosomes (i.e., located to equalize inter-marker physical distances; Fig. 1).

We extracted DNA from 189 RILs and both parental genotypes using the CTAB procedure described in [25]. RILs were then genotyped using the GoldenGate SNP typing assay (http://www.illumina.com/technology/goldengate_genotyping_assay.ilmn). The SNP typing reactions were conducted by the UTSW Genomics and Microarray Core Facility at the UT Southwestern Medical Center (Dallas, TX). The parental SNP genotypes matched the predicted nucleotides given their respective genome sequence for all markers.

### Phenotyping of RILs

All RILs, as well as parental SG and Cvi-0 plants, were grown from seed in 500 ml pots filled with a 1∶1∶1 (v/v) mixture of vermiculite, gravel, and Turface (Profile Products, Buffalo Grove, IL). Imbibed seeds were maintained at 4°C for 4 d to promote uniform germination. Over the course of the whole experiment, four growth chambers (Conviron BDR16, Winnipeg, CAN) were used and each involved both a 380 ppm (current) and 700 ppm (elevated) [CO_2_] treatment. At any given time, two chambers were controlled at 380 ppm and two were controlled at 700 ppm CO_2_, and plants remained in the same chamber throughout their life cycle. Six replicates (n = 6) were grown for each RIL line at each [CO_2_] treatment, and these were distributed randomly among chambers. Light levels were maintained at ∼800 µmol m^−2^ s^−1^ with a 14/10 h photoperiod (*Arabidopsis* is a long-day species). Temperatures were maintained at 25/18°C (day/night) with relative humidity of 60/90%. Parental genotypes and RILs did not require vernalization to initiate flowering. All plants were watered to saturation twice daily and received one dose of half-strength Hoagland's solution each morning during watering. We recorded time to visible flowering (defined as the main inflorescence being 1 cm in length) every 24 hr, and flowering time was determined from the average of 6 replicates within each RIL line. Leaf number at flowering was also measured, which serves as a proxy for plant developmental stage at flowering [26].

### QTL mapping

We used AntMap version 1.2 [27] to construct the linkage map (see Figure 1). For this, we applied the RI option treating heterozygotes as missing data. The ordering of markers based on recombination in the RIL set was fully consistent with genomic locations of the SNPs. The linkage map included 163 loci. The deficiency (relative to the intended 192) is due to genotyping failure for 17 markers and because 12 markers exhibited complete segregation distortion (all RILs fixed for the Cvi-0 marker allele). The latter group consisted of two sets, four contiguous markers on chromosome 2 and eight contiguous markers on chromosome 3. For polymorphic markers, segregation distortion was significant but not typically favoring the Cvi-0 allele.

We mapped QTL using the composite interval mapping (CIM) function of Windows QTL Cartographer 2.0 [28] with default model settings (forward regression method with five control markers, a window size of 10 cM, and a walk speed of 2 cM). Genome-wide threshold values were established for each trait (at a p-value ≤0.05 significance level) using 1000 permutations of the phenotypes against the genotypes [28], [29]. We used the S7 option to allow QTL estimation with heterozygotes. The phenotype for each RIL was the difference in days to flower (*ln*-transformed) between the 380 and 700 ppm CO_2_ treatments. We also mapped QTL for days to flower within each treatment (see Figures S1, S2).

### Statistical analysis of phenotypic responses

In order to assess the effects of [CO_2_] on flowering time within the parental genotypes, we conducted t-tests to contrast plants grown at different [CO_2_] treatments (within genotypes). To assess the response of RILs for flowering time and leaf number at flowering, we applied an ANOVA with RIL, [CO_2_], and chamber as main effects, and included an interaction of RIL with [CO_2_]. Chamber and RIL were treated as random effects, while [CO_2_] was treated as fixed. The raw measurements were *ln*-transformed to reduce heteroscedasticity.

### Mutant analysis

To identify possible candidate genes in *Arabidopsis*, we conducted a mutant analysis focusing on the flowering time genes (see description of marker choice above) that were associated with the significant QTL found on chromosome 1 (see Results). We tested two homozygous T-DNA knock-out mutants (developed by the Salk Institute for Biological Studies) and three EMS mutants that were available from the *Arabidopsis* Biological Resource Center (The Ohio State University), as well as their associated wild-types including: CRYPTOCHROME 2 (CRY2), GIBBERELLIN 2-OXIDASE, PHYTOCHROME A (PHYA), GIBBERELLIC ACID INSENSITIVE (GAI) and MOTHER OF FT AND TFL1 (MFT). The mutants for CRY2, GAI and PHYA were in the Landsberg erecta (Ler-0) background, and the mutants for MFT and GIBBERELLIN 2-OXIDASE were in the Columbia (Col-0) background. Wild-types were grown side-by-side with mutant plants. Plants (n = 15 per genotype) were grown in growth chambers maintained at either 380 or 700 ppm [CO_2_], with similar growing conditions as in the phenotyping study used for the RILs (described above). Flowering time in response to [CO_2_] was recorded for all plants as described above. We determined the effects of [CO_2_] on each flowering time mutant by applying ANOVAs with genotype (includes mutant and corresponding wild-type) and [CO_2_] as main effects (with their interaction), and flowering time as the dependent variable.

## Results

**Figure 2:**
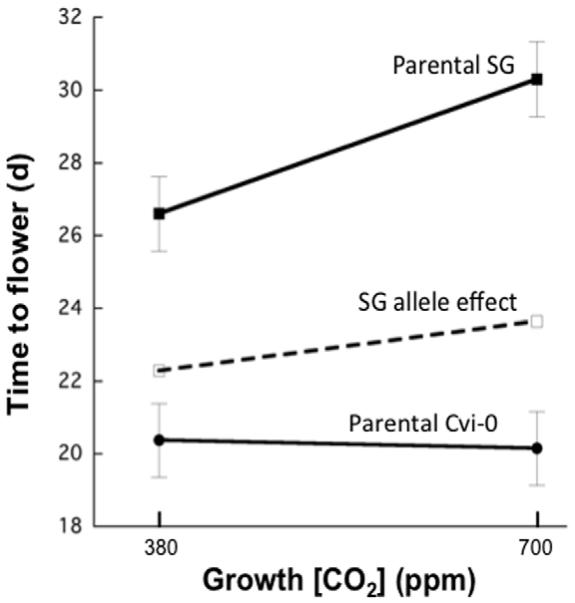
Time to flower at 380 and 700 ppm CO_2_ for parental genotypes (SG and Cvi-0), as well as the predicted effect of the SG allele (significant QTL on chromosome 1) on flowering time in Cvi-0 (determined through RIL responses with the SG allele). Symbols are means ±1 standard error.

The parental genotypes grown simultaneously with the RILs showed the expected phenotypic responses (Fig. 2), whereby Cvi-0 flowered earlier than SG, and Cvi-0 did not show statistical differences in flowering time between 380 and 700 ppm CO_2_. In contrast, SG flowered significantly later at elevated [CO_2_] compared with current [CO_2_] (t_[58]_  = 3.33, p = 0.002; Fig. 2).

**Figure 3:**
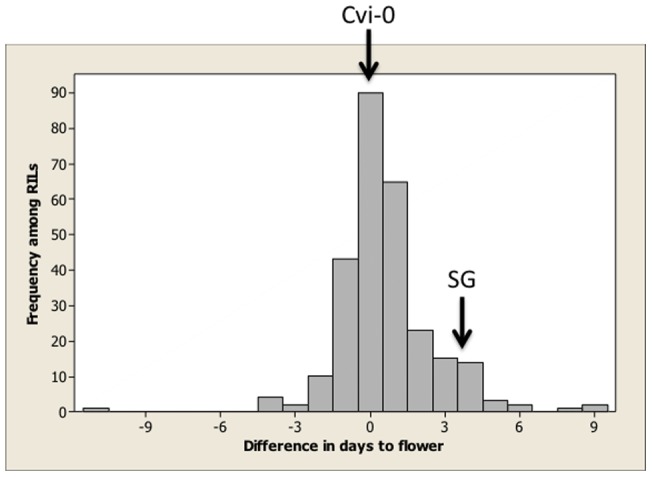
Frequency distribution of RIL responses for flowering time. The x-axis was calculated as days to flower at 700 ppm [CO_2_] minus days to flower 380 ppm [CO_2_]. Thus, positive values represent delays in flowering at elevated [CO_2_] and negative values represent more rapid flowering (with 0 being no change in flowering time due to [CO_2_]). Differences for parental Cvi-0 and SG are shown with arrows.

For the RIL analysis (parents were excluded), there were highly significant effects of RIL, chamber, and growth [CO_2_] on time to flower. Because we distributed replicates across four chambers, and because each chamber was used for both a 380 and 700 ppm round (in alternating order), the effects of chamber were effectively distinguished from those of RIL and [CO_2_] treatments. Importantly, there was a highly significant interaction between RIL and growth [CO_2_] for time to flower (p<0.001), indicating that RILs responded differently to elevated [CO_2_]. Direct inspection revealed an abundance of responses in both directions, with some RILs flowering earlier and some flowering later at elevated [CO_2_] (Fig. 3). The responses of RILs relative to the parents indicate transgressive segregation for both earlier and later flowering (Fig. 3).

**Figure 4:**
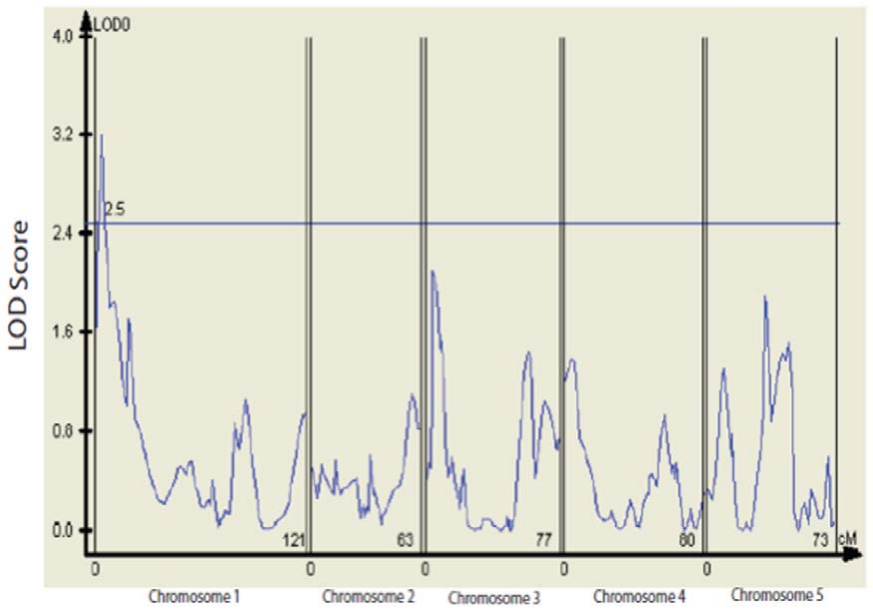
The LOD score as a function of map location. The horizontal line at 2.5 is the significance threshold.

Composite Interval Mapping identified only a single major QTL for the differential response of flowering time at elevated [CO_2_] on chromosome 1 (Fig. 4). A genome-wide threshold for significant LOD values was established at 2.5 by permutation. An interval based on the 2-LOD drop criterion for spatially locating the QTL starts at map location 0.0 and ends at location 26.0 (ending between markers m14 and m15) of chromosome 1. This QTL accounts for approximately 1/3 of the difference in flowering time between 380 and 700 ppm CO_2_ between the parental genotypes (SG and Cvi-0), and therefore can be considered a QTL of major effect. The QTL mapping results for *ln*-days to flower within each [CO_2_] level are reported as Figures S1, S2.

The marker most near the significant QTL peak, AT1G04400, is a flowering time candidate known as *CRYPTOCHROME 2* (*CRY2*). Other nearby flowering candidates include GIBBERELLIN 2-OXIDASE, PHYTOCHROME A (PHYA), GIBBERELLIC ACID INSENSITIVE (GAI) and MOTHER OF FT AND TFL1 (MFT). The estimated effect of the SG allele at the significant QTL on chromosome 1 is illustrated in Figure 2. The dashed line shows the predicted effect on flowering time when substituting the SG allele for the Cvi-0 allele (both homozygous) at the AT1G04400 marker locus. This allele would be predicted to increase the time to flowering in the Cvi-0 genotype by 1.9 d at 380 ppm CO_2_ and by 3.5 d at 700 ppm CO_2_ (accounting for a large portion of the differential response). Furthermore, RILs containing the Cvi-0 allele at the AT1G04400 marker locus do not show flowering time differences between [CO_2_] treatments (p>0.25, data not shown).

There was a strong positive genetic correlation (r = 0.81, p<0.001) between days to flower and number of rosette leaves at flowering when including all RILs at both [CO_2_] treatments (data not shown). On average, RILs with the SG allele at the significant QTL had more than twice the leaf number at flowering (Mean ± SE: 31±1, n = 130) compared to RILs with the Cvi-0 allele (Mean ± SE: 14.3±0.3, n = 220). Furthermore, genotype (SG versus Cvi-0) had a significant effect on the difference in leaf number at flowering between 700 and 380 ppm CO_2_ (p<0.0001). On average, RILs with the SG allele had 9±1 more leaves (n = 65) when initiating flowering at 700 versus 380 ppm CO_2_, whereas RILs with the Cvi-0 allele showed only a modest average increase in leaf number between 700 and 380 ppm CO_2_ (1.4±0.3 more leaves; n = 110).

**Figure 5:**
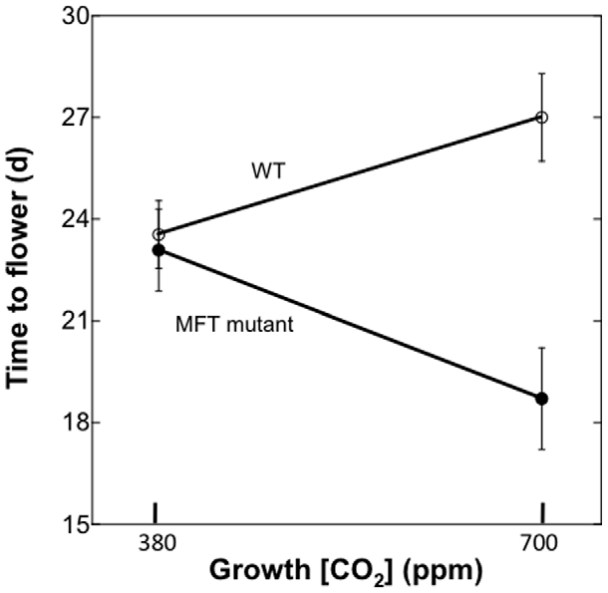
Response of the MFT knock-out mutant and wild-type (WT), which is Columbia (Col-0) to current (380 ppm) and elevated (700 ppm) CO_2_. Symbols are means ± 1 standard error.

For the mutant analysis with flowering time genes under the significant QTL, a difference in the CO_2_ response between mutant and wild-type plants would be indicative of a potential gene candidate. In other words, a significant interaction (CO_2_ x genotype) for flowering time would suggest that gene action at a locus is sensitive to elevated [CO_2_] (regardless of the direction of the response), and our QTL analysis greatly reduced the pool of possible flowering time candidates. Of the five mutants analyzed (see Methods), only MFT showed a significant CO_2_ x genotype (mutant and wild-type) interaction (p = 0.003) for flowering time. Post-hoc analysis did not reveal a significant difference between the MFT mutant and Col-0 wild-type at 380 ppm [CO_2_], but these plants showed major differences in flowering time at 700 ppm [CO_2_] (P = 0.0001), with the MFT mutant flowering much earlier at 700 ppm [CO_2_], while the wild-type delayed flowering (Fig. 5). In addition, there was not a significant CO_2_ × genotype interaction between the MFT mutant and Col-0 wild-type for number of rosette leaves at flowering.

For flowering time, PHYA showed no significant effects of [CO_2_], genotype or the interaction, and GAI only showed a genotype effect, but was not responsive to [CO_2_]. CRY2 and GIBBERELLIN 2-OXIDASE only showed an effect of [CO_2_], and therefore those mutants did not differ from their respective wild-types in response to elevated [CO_2_].

## Discussion

A number of studies have identified both major and minor QTL affecting flowering time in *Arabidopsis thaliana* ([30]–[37]), as well as in other wild [38], [39] and crop species [40]–[42]. Of these studies, several have included environmental manipulations, with the majority focusing on changes in photoperiod (e.g., long versus short days; [31], [43]) and/or exposure to cold temperatures (e.g., vernalization [32]). Although these are important issues, there has been a major gap in discovery of QTL that affect flowering time under conditions most relevant to global change that involves future environments. It is clear that rising [CO_2_] will have a major impact on flowering time, and that this effect may be as pronounced as increasing temperatures [11], [12]. Thus, it is critical to understand the influence of elevated [CO_2_] on flowering time at the genomic level in order to more accurately predict phenological shifts in response to global change and to best select crops for future environments.

In this paper, we present the first QTL study to investigate the effects of increasing [CO_2_] on flowering time. We developed a mapping population that included the Cvi-0 genotype of *Arabidopsis*, which has been used in several other QTL-flowering time studies [30], [32], [37], [44], and that we found to be unaffected by [CO_2_] for flowering time [4], [20]. The other parental genotype, SG, was previously adapted to elevated [CO_2_] through artificial selection [17], [18] and was fully sequenced using the Illumina platform for this study. SG was important to include because it was previously found to exhibit the most pronounced response to selection for high seed number (fitness) at elevated [CO_2_] (700 ppm), and at the same time its developmental patterns ran counter to the majority of other genotypes that were selected at elevated [CO_2_] [18]. SG exhibited delayed flowering at elevated [CO_2_], whereas the majority of other selected genotypes exhibited accelerated flowering [17]. It is also important to point out that in the original selection experiment, SG was a product of an initial cross between a field-collected genotype from Poland (CS3177, at the ABRC) and Seattle, Washington (CS6187), and was selected for high seed number at elevated [CO_2_] through variation resulting from recombination and segregation over five generations. Thus, SG is the product of segregation from a single heterozygous plant.

In this study, we focused on identifying QTL that contribute to differential flowering time at predicted future (700 ppm) versus current (380 ppm) [CO_2_]. The phenotype applied to the QTL analysis was the *difference* in flowering times between these [CO_2_] treatments. We identified only one significant QTL for differential flowering time at elevated [CO_2_]. This QTL is in the upper region of chromosome 1 with a peak nearest to marker AT1G04400. This was a major-effect QTL in that it explained approximately 1/3 of the differential response of flowering time at elevated [CO_2_] between the parental genotypes (Fig. 2). Thus, the main changes in flowering time at elevated [CO_2_] are likely the result of polymorphism in one or a small number of genes, by virtue of the fact that we found only one significant QTL in this case. Perhaps most importantly, the SG allele at this QTL delays flowering time to a greater extent at 700 versus 380 ppm CO_2_ (Fig. 2), providing a genetic basis for the improved fitness of this genotype at elevated [CO_2_]. The remaining variation was likely due to many minor QTLs with effects lesser than the detection limits of this study.

There are a number of candidate genes in the vicinity of the significant QTL on chromosome 1 that could be driving differential flowering time at elevated [CO_2_]. Candidates include genes related directly to flowering time, those that sense photoperiod, those that govern the meristem transition from the vegetative to reproductive states, genes that are involved in sugar sensing, and those that control inflorescence development and overall growth form. With respect to flowering time genes, some predictions can be made at present through our mutant analysis and based on work by others. Importantly, several previous studies using Cvi-0 as a parent mapped a flowering time QTL to the upper portion of chromosome 1 [32], [43], in the vicinity of our QTL. This region includes *CRY2* (*CRYPTOCHROME 2*), a blue (and red) light-dependent cryptochrome that senses long days. El-Assal and colleagues [43] determined that Cvi-0 harbors a mutation in *CRY2* that confers early flowering. This same mutation may explain a portion of the overall earlier flowering response of Cvi-0 observed in our study. However, the Cvi-0 allele of *CRY2* does not explain the differential flowering time feature of our QTL. This is mainly because RILs harboring the Cvi-0 allele at the significant QTL did not show flowering time differences between [CO_2_] treatments (see Results).

It may be that the previously unmapped SG allele of *CRY2* is responsive to [CO_2_], generating the differential flowering time of our QTL. It is important to note, however, that in out mutant analysis, we did not find a significant [CO_2_] × genotype interaction for CRY2. Knock-out of this gene in the Ler-0 background does not affect flowering time in response to elevated [CO_2_]. There are, however, other candidate genes in the upper portion of chromosome 1, including the phytochrome, *PHYA,* which influence flowering time through similar mechanisms [45]. Photoreceptors detect day length in the leaves, and this response is then transferred to the shoot apical meristem through *Flowering Locus T* (*FT*). In addition, recent work by Song *et* *al*. [14] suggests that photoreceptors like *PHYA* and *CRY2* may interact with CO_2_ in affecting flowering time through a mechanism that is not yet understood. However, preliminary evidence against photoreceptor candidates in our QTL study comes from the previous finding that SG exhibits later flowering at elevated [CO_2_] through delays in the down-regulation of *FLC* [*18*]. The newest models of flowering pathways indicate that photoreceptors influence flowering time through pathways that are mainly independent of *FLC* (Wellmer & Riechmann 2010), suggesting that photoreceptor genes are less likely to play a role in controlling differential flowering time at elevated [CO_2_] in our system. In addition, PHYA also did not yield a significant [CO_2_] × genotype interaction in our mutant analysis.

Our mutant analysis indicated that MFT may be a candidate gene for altered flowering time at elevated [CO_2_]. Here we found that wild-type Col-0 delayed flowering between 380 and 700 ppm CO_2_, as has been observed in other studies (e.g., [46]). In contrast, MFT mutant plants exhibited a major acceleration in flowering time at elevated [CO_2_]. In this study, we were looking for mutants that responded differently to elevated [CO_2_] relative to corresponding wild-type plants, indicating a possible role for that gene in influencing flowering time at elevated [CO_2_]. The direction of such a response may not necessarily be delayed as in SG, because both the MFT alleles and genetic backgrounds are different in the QTL mapping and mutant analysis. Interestingly, Yoo *et* *al*. [47] found that over-expression of MFT accelerated the flowering time of *Arabidopsis* in a modern [CO_2_] environment, whereas the knock-out mutant was aphenotypic. In our case, we found that the knock-out mutant also responded similarly to wild-type at 380 ppm CO_2_, although it showed a major acceleration in flowering time at elevated [CO_2_] (Fig. 5). Currently, the full function of MFT is not well understood and the mechanisms accounting for its influence on flowering time are being investigated (e.g., [48]). The consideration of the CO_2_ response into future MFT studies may prove interesting. Furthermore, these results beg the question of how relevant mutant studies will be in the future as [CO_2_] continues to rise, since the majority of these studies are conducted at current [CO_2_].

Other possible gene candidates come from a study using the Landsberg *erecta* X Kondara *Arabidopsis* mapping population [49]. These authors mapped a number of flowering time QTL to the upper section of chromosome 1, including genes affecting sugar concentrations of shoot tissue (mainly glucose and fructose). These QTL are tempting candidates given that elevated [CO_2_] increases sugar concentrations of leaves and sugar sensing can influence flowering times [11], [18], [50], [51]. Other flowering time QTL altered plant size at flowering, mainly through plant height (El-Lithy *et* *al.*, 2010). Although we did not specifically measure this trait, we did find shifts towards greater rosette leaf number at flowering for RILs containing the SG allele, which are RILs that also delayed flowering at elevated [CO_2_]. This indicates that our significant QTL not only delays flowering time at elevated [CO_2_], but also alters the stage at which flowering occurs.

In summary, we have conducted the first QTL study to investigate the effects of elevated [CO_2_] on flowering time. We made use of the powerful tools in the *Arabidopsis* model system, as well as incorporating a parental genotype that was previously adapted to elevated [CO_2_] in our laboratory. We found one significant QTL of major-effect on chromosome 1 that explained approximately 1/3 of the difference in flowering time between parental genotypes grown at current versus elevated [CO_2_]. In addition, this QTL influenced the stage at which flowering occurred, as determined from shifts in rosette leaf number at flowering under elevated [CO_2_]. In a mutant analysis with flowering time genes under the significant QTL, we determined that MFT is a possible flowering gene candidate with knock-out plants exhibiting altered flowering time at elevated [CO_2_]. This study sheds light on the underlying genetic architecture that controls flowering time shifts at elevated [CO_2_]. Such genomic information will be critical for predicting phenological shifts that will accompany global change events predicted for the near future, and will be necessary for crop improvements as [CO_2_] continues to rise.

### Supporting Information

The QTL mapping results for flowering time (*ln* days to flower) within each [CO_2_] treatment are reported as Figures S1 and S2 below. The conventions for these figures are the same as for Figure 4 in the main text. The significance threshold for LOD scores was determined by permutation for each trait. There are three significant QTL at 380 ppm CO_2_, one each on chromosomes 1, 3, and 5 (Figure S1). There are also three significant QTL at 700 ppm CO_2_ (Figure S2). Two of the three co-localize with 380 ppm CO_2_, although the estimated location of the chromosome 5 QTL is slightly different. Importantly, the same genomic region responsible for the differential response (Figure 4 of main text) is also the major QTL for flowering time within [CO_2_] environments.

## Supporting Information

Figure S1 Top: LOD score for *ln*-days to flower as a function of map position (chromosomes 1–5 distinguished by vertical double lines) for RILs grown at 380 ppm CO_2_. Bottom: Estimated additive effect of QTL as a function of map position.
(TIFF)

Figure S2 Top: LOD score for *ln*-days to flower as a function of map position (chromosomes 1–5 distinguished by vertical double lines) for RILs grown at 700 ppm CO_2_. Bottom: Estimated additive effect of QTL as a function of map position. Vertical scale in each panel is different than in Figure S1.
(TIFF)

Table S1 Flowering time genes are identified in the first column with associated genomic location and name in the map of Figure 1.
(DOC)
