# Supplementary figures and images for: Identification of a Major QTL That Alters Flowering Time at Elevated [CO2] in Arabidopsis thaliana
Source: PLoS One. 2012 Nov 21;7(11):e49028. doi: 10.1371/journal.pone.0049028 (PMC3504057; doi:10.1371/journal.pone.0049028)

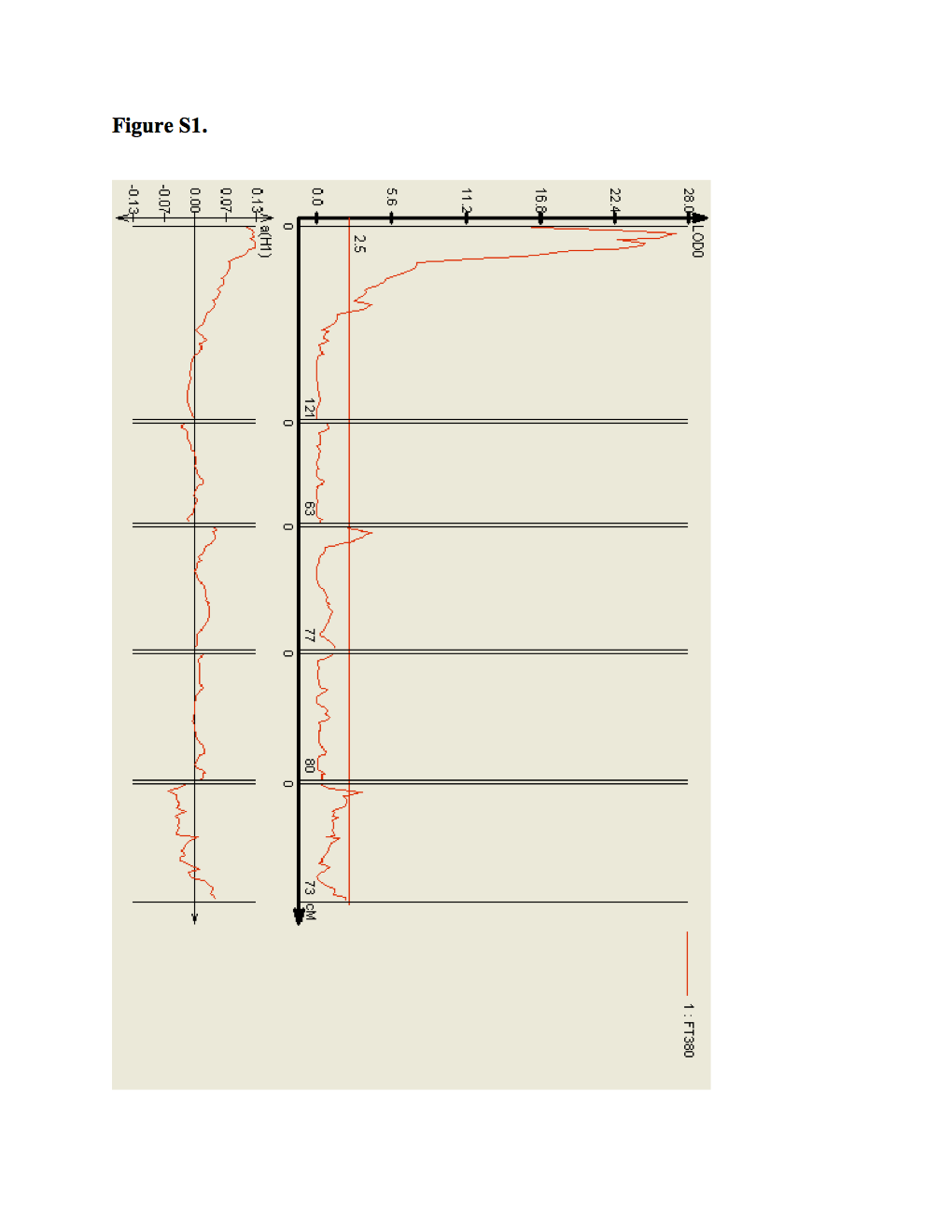

Supplement: Figure S1 — Top: LOD score for ln-days to flower as a function of map position (chromosomes 1–5 distinguished by vertical double lines) for RILs grown at 380 ppm CO2. Bottom: Estimated additive effect of QTL as a function of map position. (TIFF) [file pone.0049028.s001.tif]

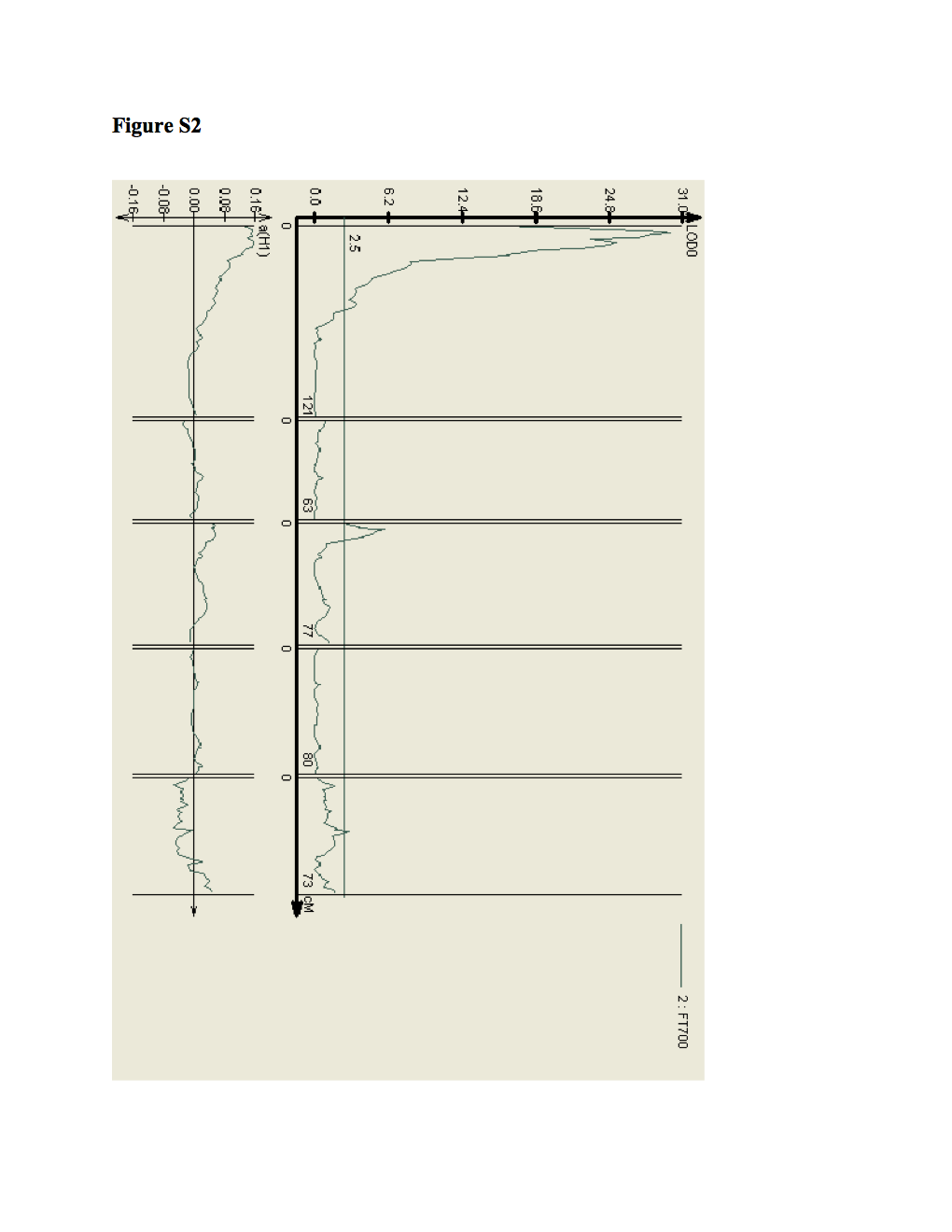

Supplement: Figure S2 — Top: LOD score for ln-days to flower as a function of map position (chromosomes 1–5 distinguished by vertical double lines) for RILs grown at 700 ppm CO2. Bottom: Estimated additive effect of QTL as a function of map position. Vertical scale in each panel is different than in Figure S1. (TIFF) [file pone.0049028.s002.tif]
