# Supplementary material for: Identification of a Major QTL That Alters Flowering Time at Elevated [CO2] in Arabidopsis thaliana
Source: PLoS One. 2012 Nov 21;7(11):e49028. doi: 10.1371/journal.pone.0049028 (PMC3504057; doi:10.1371/journal.pone.0049028)
Supplement: Table S1 — Flowering time genes are identified in the first column with associated genomic location and name in the map of Figure 1. (DOC) [file pone.0049028.s003.doc]

**Table S1.**

| **Flowering time gene** | **Chromosome** | **Coordinate** | **Marker name in map** |
| --- | --- | --- | --- |
| GI | 1 | 64149 | m1 |
| RGA | 1 | 482966 | m2 |
| AT1G04400 | 1 | 1193671 | m3 |
| PHYA | 1 | 3088452 | m6 |
| GAI | 1 | 5154269 | m9 |
| MFT | 1 | 6260820 | m12 |
| FRL2 | 1 | 11411547 | m18 |
| FT | 1 | 24339025 | m32 |
| RGL1 | 1 | 24701036 | m33 |
| FKF1 | 1 | 25555428 | m34 |
| FDP | 2 | 7690796 | m48 |
| PHYB | 2 | 8153157 | m51 |
| FVE | 2 | 8425814 | m52 |
| SVP | 2 | 9392344 | m55 |
| ATC | 2 | 11726414 | m60 |
| AT2G39810 | 2 | 16583724 | m68 |
| SOC1 | 2 | 18796279 | m72 |
| RGL2 | 3 | 777885 | m78 |
| FLK | 3 | 1259002 | m80 |
| GAr2 | 3 | 1449443 | m82 |
| SPY | 3 | 3634241 | m86 |
| GAr3 | 3 | 23303380 | m117 |
| FRI | 4 | 70933 | m119 |
| LD | 4 | 1082295 | m121 |
| GA1 | 4 | 1226169 | m122 |
| CRY1 | 4 | 5730481 | m129 |
| ESD4 | 4 | 8988991 | m134 |
| PHYD | 4 | 9220882 | m136 |
| PHYE | 4 | 10033711 | m138 |
| EBS | 4 | 11768258 | m142 |
| SLY1 | 4 | 12558804 | m144 |
| AT4G24540 | 4 | 12667743 | m145 |
| AT4G30200 | 4 | 14731531 | m148 |
| FD | 4 | 17031622 | m153 |
| TFL1 | 5 | 1044498 | m157 |
| ATMYB33 | 5 | 1779705 | m158 |
| FLC | 5 | 3173293 | m160 |
| CO | 5 | 5180322 | m162 |
| FRL1 | 5 | 5343399 | m164 |
| TFL2 | 5 | 5827398 | m165 |
| HUA2 | 5 | 7658734 | m168 |
| FPF1 | 5 | 8554370 | m170 |
| GAr1 | 5 | 9637128 | m171 |
| VIN3 | 5 | 23277596 | m185 |
| LFY | 5 | 24858665 | m188 |
| BFT | 5 | 24940219 | m189 |
| AT5G62430 | 5 | 25094696 | m190 |
| AT5G62640 | 5 | 25148846 | m191 |
